# Case Report: Breaking the diagnostic dilemma of “lymphoma-like” presentation: a case of adult-onset Still's disease characterized by generalized lymphadenopathy and extremely elevated ferritin

**DOI:** 10.3389/fmed.2026.1886283

**Published:** 2026-09-21

**Authors:** Yanhui Duan, Wei Liu, Kun Zhang, Jian Lu

**Affiliations:** Department of Infectious Diseases, Shenzhen University General Hospital, Shenzhen University, Shenzhen, Guangdong, China

**Keywords:** adult-onset Still's disease, case report, ferritin, hyperferritinemia, lymph node biopsy, lymphadenopathy, lymphoma-like presentation, PET/CT

## Abstract

Adult-onset Still's disease (AOSD) is a rare autoinflammatory disease, the clinical manifestations of which often overlap with other diseases such as lymphoma. This overlap makes differential diagnosis difficult, especially in patients with generalized lymphadenopathy, splenomegaly, and systemic metabolic hyperactivity. In this article, we discuss a 53-year-old East Asian (Chinese) female patient who had a fever, transient rash, generalized lymphadenopathy, and splenomegaly. An 18F-FDG PET/CT scan showed diffuse hypermetabolism in multiple lymph nodes, her spleen, and her bone marrow, and her serum ferritin level was elevated at 15,485 ng/mL, leading to strong suspicion of lymphoma. However, an excisional biopsy of the right inguinal lymph nodes with pathological, immunohistochemical, and T-cell receptor gene rearrangement analyses showed no evidence of clonality or lymphoma. Given the typical clinical presentation and laboratory findings, the patient was diagnosed with AOSD according to the Yamaguchi classification criteria. Glucocorticoid therapy rapidly normalized the patient's temperature, the rash disappeared, her lymph nodes decreased in size, and her ferritin and inflammation levels significantly decreased, suggesting a good therapeutic response. This case illustrates the value of lymph node biopsy in AOSD with “lymphoma-like” imaging features. Moreover, ferritin is a critical biomarker and is crucial for early identification of such difficult cases.

## 1 Introduction

Adult-onset Still's disease (AOSD) is a rare systemic autoinflammatory disorder of unknown cause. Symptoms include fevers, rashes, joint pain or arthritis, sore throat, hepatosplenomegaly, and significant elevations in peripheral white blood cells and neutrophils (1). Because there are no specific laboratory markers, diagnosing AOSD is essentially a process of exclusion, and clinicians must rule out infectious diseases, malignancies (especially lymphomas), and other conditions (2). Lymphadenopathy is common, occurring in approximately 50% of patients, is usually symmetrical, and most commonly affects the cervical region (3). However, if a patient has large multi-regional lymphadenopathy, splenomegaly, and increased metabolic activity, clinical features and imaging can easily be mistaken for lymphoma (4). Although 18F-FDG PET/CT can detect systemic metabolic activity sensitively, it is not specific enough to distinguish AOSD from lymphoma because both conditions can show diffuse high metabolic uptake in lymph nodes, the spleen, and bone marrow (5). Therefore, for patients with these “lymphoma-like” presentations, lymph node biopsy, along with pathological, immunohistochemical, and gene rearrangement analyses, is essential for differential diagnosis (6). Furthermore, in regions where tuberculosis is endemic, AOSD may be misdiagnosed as tuberculosis, which can substantially delay the correct diagnosis and treatment (7, 8).

Another important laboratory feature of AOSD is that serum ferritin is elevated several times above normal levels (9). In clinical practice, an extremely high ferritin level (usually >10,000 ng/mL) is considered an important marker of AOSD, which can be useful for distinguishing AOSD from infections and malignancies (10). Marked hyperferritinemia, however, is not specific to AOSD and may also be observed in hemophagocytic syndromes, including hemophagocytic lymphohistiocytosis (HLH) and macrophage activation syndrome (MAS) (11). However, when a very high ferritin level is associated with “lymphoma-like” imaging results, clinicians may face a significant diagnostic challenge, leading them to focus on malignancy and to delay diagnosis and treatment of AOSD. This case report describes a 53-year-old woman who had generalized lymphadenopathy, splenomegaly, diffuse hypermetabolism on PET/CT, and an extremely high ferritin level of 15,485 ng/mL, which at first strongly suggested lymphoma. However, excisional lymph node biopsy and molecular pathological tests failed to detect lymphoma, and she was ultimately diagnosed with AOSD.

## 2 Case description

### 2.1 Patient information

A 53-year-old East Asian (Chinese) woman was admitted to the Infectious Diseases Department of Shenzhen University General Hospital in November 2025 with a fever and rash lasting more than 20 days. Her past medical history included a suspected dog scratch without bleeding 6 months earlier, followed by a rash that resolved after approximately 10 days of antihistamine treatment, hysterectomy for uterine fibroids in 2023, and laparoscopic cholecystectomy for cholelithiasis in 2024. She had no history of hypertension, diabetes, or cardiac disease; no known drug or food allergies; and no prior similar febrile episodes. The fever began suddenly without a trigger, reached 39.6 °C, occurred mostly at night, and was accompanied by chills. She also had diffuse, pruritic erythematous rashes over her trunk and extremities. COVID-19 was diagnosed at the referral hospital on the basis of a positive SARS-CoV-2 antigen test; she received nirmatrelvir/ritonavir and a short course of methylprednisolone. Her fever improved temporarily, but the rash persisted and the symptoms recurred after withdrawal of treatment. She also had a sore throat, dry cough, fatigue, and nausea. Chest CT at the referral hospital showed no pneumonia or pulmonary infiltration. Empirical antibiotic therapy with cefoperazone-sulbactam was administered for approximately 1 week because bacterial infection could not be initially excluded, but it led to little clinical improvement.

### 2.2 Clinical findings

The patient had a body temperature of 38.4 °C, a heart rate of 117 beats/min, and a blood pressure of 120/86 mmHg; her body weight was 65 kg and her height was 163 cm. Patchy rashes with scratch marks were seen (Figure 1A). Multiple superficial lymph nodes were palpable in the neck, axillary, and groin areas (from pea to peanut size), with soft texture, good mobility, and no tenderness. Cardiopulmonary and abdominal examinations were unremarkable. No signs of arthritis were observed during this hospital stay.

**Figure 1:**
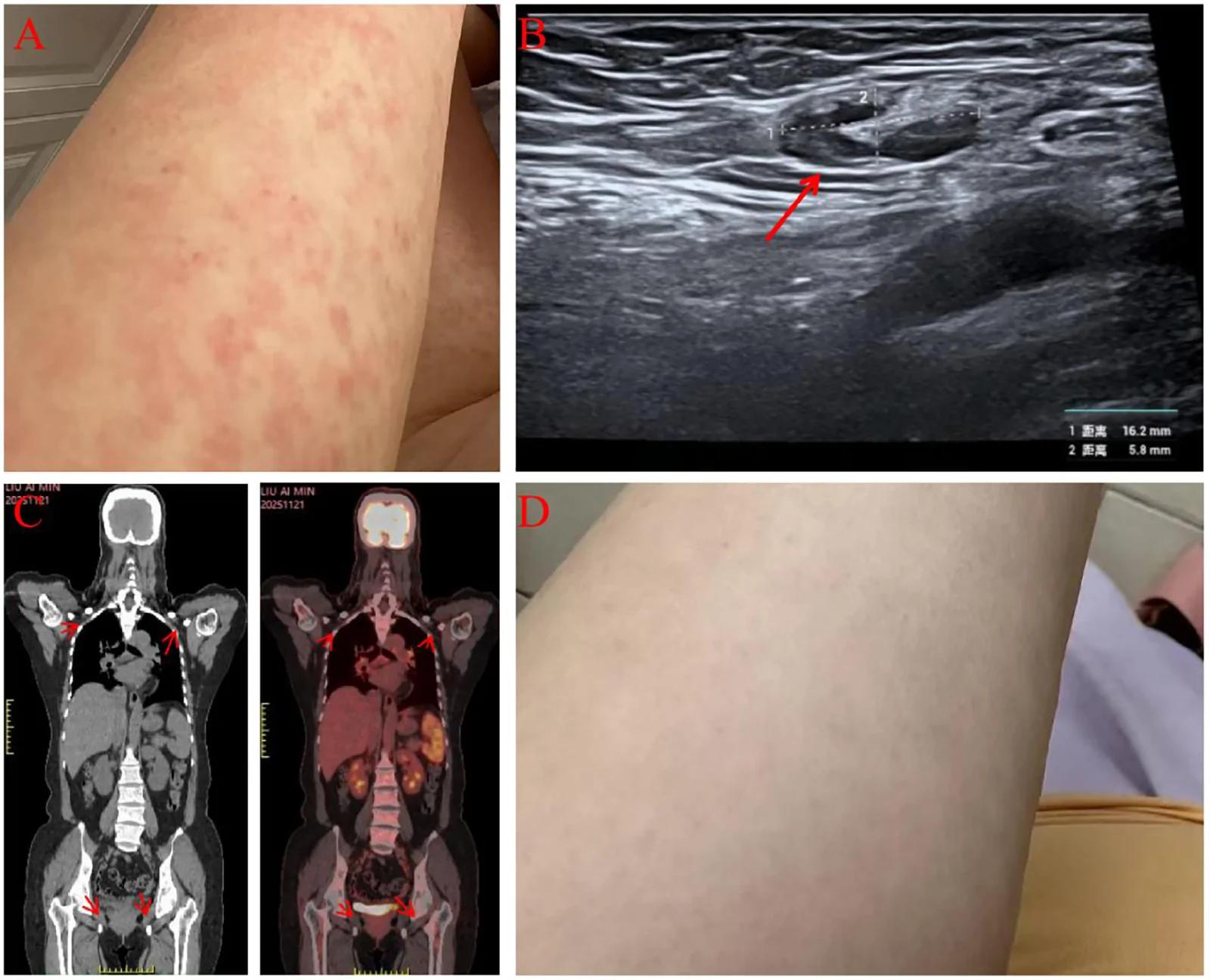
Clinical and imaging findings. **(A)** Diffuse erythematous rash at admission. **(B)** Superficial lymph node ultrasonography shows enlarged lymph nodes. **(C)** Whole-body PET/CT maximum-intensity projection shows generalized hypermetabolic lymphadenopathy, splenomegaly, and diffuse bone marrow metabolic activation. **(D)** Regression of rash after glucocorticoid therapy.

### 2.3 Diagnostic assessment

Laboratory investigations showed mild leukocytosis that had been reported at the referral hospital, neutrophilia with a decreased lymphocyte percentage (14.7%; absolute count 0.89 × 10^⁹^/L), mild anemia (hemoglobin 102 g/L), markedly elevated inflammatory markers (high-sensitivity C-reactive protein 43.04 mg/L, serum amyloid A 92.83 mg/L, interleukin-6 22.27 pg/mL, and procalcitonin 0.514 ng/mL), an elevated erythrocyte sedimentation rate (114 mm/h), and severe hyperferritinemia, with a peak serum ferritin of 15,485 ng/mL (reference range, 13–150 ng/mL). Liver function tests showed elevated aspartate aminotransferase (AST; 42 U/L) and gamma-glutamyl transferase (GGT; 204 U/L), with decreased albumin (35 g/L). The complete laboratory findings are summarized in Table 1. These findings indicated severe systemic inflammation but were insufficient to distinguish AOSD from infection or hematologic disease (Figure 1B). Whole-body 18F-FDG PET/CT showed increased metabolic activity in multiple lymph nodes (cervical, axillary, mediastinal, abdominal, and inguinal regions), splenomegaly, and diffuse bone marrow metabolic activity (Figure 1C). Given the patient’s fever, generalized lymphadenopathy, splenomegaly, and increased FDG uptake, lymphoma was a primary differential diagnosis. To determine the nature of the lymphadenopathy, an excisional biopsy of a right inguinal lymph node was performed. This nodal group was selected because the nodes were large, superficial, and readily accessible, with a low procedural risk. Although cervical or axillary nodes may provide richer morphological information, the inguinal node yielded adequate tissue for histopathological, immunohistochemical, and gene rearrangement analyses. Histopathology showed reactive lymphoid hyperplasia. Immunohistochemistry showed a mixed lymphoid pattern with CD3-positive T-cell areas, CD20-positive B-cell areas, and CD21-positive follicular dendritic cell networks, with Ki-67 staining indicating a reactive process (Figure 2). The T-cell receptor gene rearrangement analysis showed no evidence of clonality or lymphoma. Collectively, these findings provided no evidence of lymphoma. Infection-related tests showed no evidence of an active infection: blood cultures (two sets, aerobic and anaerobic) were negative, pathogen metagenomic next-generation sequencing detected no significant pathogen, serology for Epstein–Barr virus (IgM negative; VCA-IgG and EBNA-IgG positive, indicating past infection) and cytomegalovirus (IgM negative; IgG positive, indicating a past infection) showed no active infection, and hepatitis B surface antigen was negative (with positive anti-HBs, anti-HBc and anti-HBe, consistent with a past infection). In addition, hepatitis C antibody, HIV antigen/antibody, and *Treponema pallidum* antibody tests were all negative. Tuberculosis was screened for using pathogen metagenomic next-generation sequencing, which detected no significant pathogens, including *Mycobacterium tuberculosis*. Autoimmune screening, including antinuclear antibodies (ANA), a 15-antigen ANA panel, and anti-neutrophil cytoplasmic antibodies (ANCA, c-ANCA, and *p*-ANCA), was negative, supporting the exclusion of systemic lupus erythematosus and ANCA-associated vasculitis. After excluding infection, lymphoma, and other disorders, AOSD was diagnosed based on the presence of intermittent high-grade fever, rash, sore throat, neutrophilic inflammation, hepatic involvement, generalized lymphadenopathy, splenomegaly, and hyperferritinemia. The patient fulfilled the Yamaguchi classification criteria for AOSD, satisfying two major criteria (intermittent high-spiking fever of ≥39 °C lasting more than 1 week and a typical evanescent rash) and four minor criteria (sore throat, generalized lymphadenopathy and/or splenomegaly, abnormal liver function tests, and negative rheumatoid factor and ANA) (12). Although mild leukocytosis had been reported at the referral hospital, the patient’s highest white blood cell count during our hospitalization was 9.45 × 10^⁹^/L, which did not reach the threshold of 10,000/µL. The diagnostic criteria and the features fulfilled by this patient are summarized in Table 2. The Fautrel criteria could not be applied because glycosylated ferritin was not measured in this patient (13).

**Table 1:** Laboratory findings at admission and during follow-up.

Parameter	Admission value	Reference range	Peak value	Follow-up/outcome
White blood cell count	6.05 × 10^⁹^/L	4.0–10.0 × 10^⁹^/L	9.45 × 10^⁹^/L (day 7)	8.26 × 10^⁹^/L (day 22)
Neutrophils	76.6%	40%–70%	81.2%	70.1%
Lymphocytes	14.7% (0.89 × 10^⁹^/L)	20%–40%	–	12.6%
Hemoglobin	102 g/L	115–150 g/L	102 g/L (lowest)	121 g/L; 136 g/L at follow-up
Platelets	249 × 10^⁹^/L	125–350 × 10^⁹^/L	379 × 10^⁹^/L	243 × 10^⁹^/L
Ferritin	15,485 ng/mL	13–150 ng/mL	15,485 ng/mL	8,496→2,070→1,438→712 ng/mL; 318 ng/mL at follow-up
High-sensitivity C-reactive protein	43.04 mg/L	<3 mg/L	74.97 mg/L (day 3)	0.28 mg/L (day 22)
Procalcitonin	0.514 ng/mL	<0.05 ng/mL	0.514 ng/mL	<0.020 ng/mL
Serum amyloid A	92.83 mg/L	<10 mg/L	165.08 mg/L (day 3)	–
Interleukin-6	22.27 pg/mL	<7 pg/mL	23.65 pg/mL (day 3)	–
Lactate dehydrogenase	394 U/L	120–250 U/L	394 U/L	–
Glycosylated ferritin	Not performed	<20%	–	–
Soluble IL-2 receptor	Not performed	Not performed	–	–
Alanine aminotransferase	Within normal range at admission^a^	7–40 U/L	169 U/L (day 17)	28 U/L at follow-up
Aspartate aminotransferase	42 U/L	13–35 U/L	67 U/L	–
Gamma-glutamyl transferase	204 U/L	7–45 U/L	273 U/L (day 17)	56 U/L at follow-up
Albumin	35 g/L	35–52 g/L	–	43 g/L
Triglycerides	1.92 mmol/L	<1.7 mmol/L	1.92 mmol/L	–
Fibrinogen	5.26 g/L	2.0–4.0 g/L	5.26 g/L	–
D-dimer	5.23 mg/L	<0.5 mg/L	5.23 mg/L	0.79 mg/L
NK-cell activity	Not performed	Not performed	–	–
Antinuclear antibodies	Negative	Negative	–	–
Anti-neutrophil cytoplasmic antibodies	Negative	Negative	–	–

Reference ranges are commonly used adult values and should be confirmed against the local laboratory ranges. Laboratory values were obtained from the patient's original medical records.

a Alanine aminotransferase was not flagged as abnormal on admission and is shown as within normal range; it became elevated from day 7 of hospitalization. Glycosylated ferritin, soluble IL-2 receptor, NK-cell activity, and bone marrow examination were not performed during the hospitalization.

**Figure 2:**
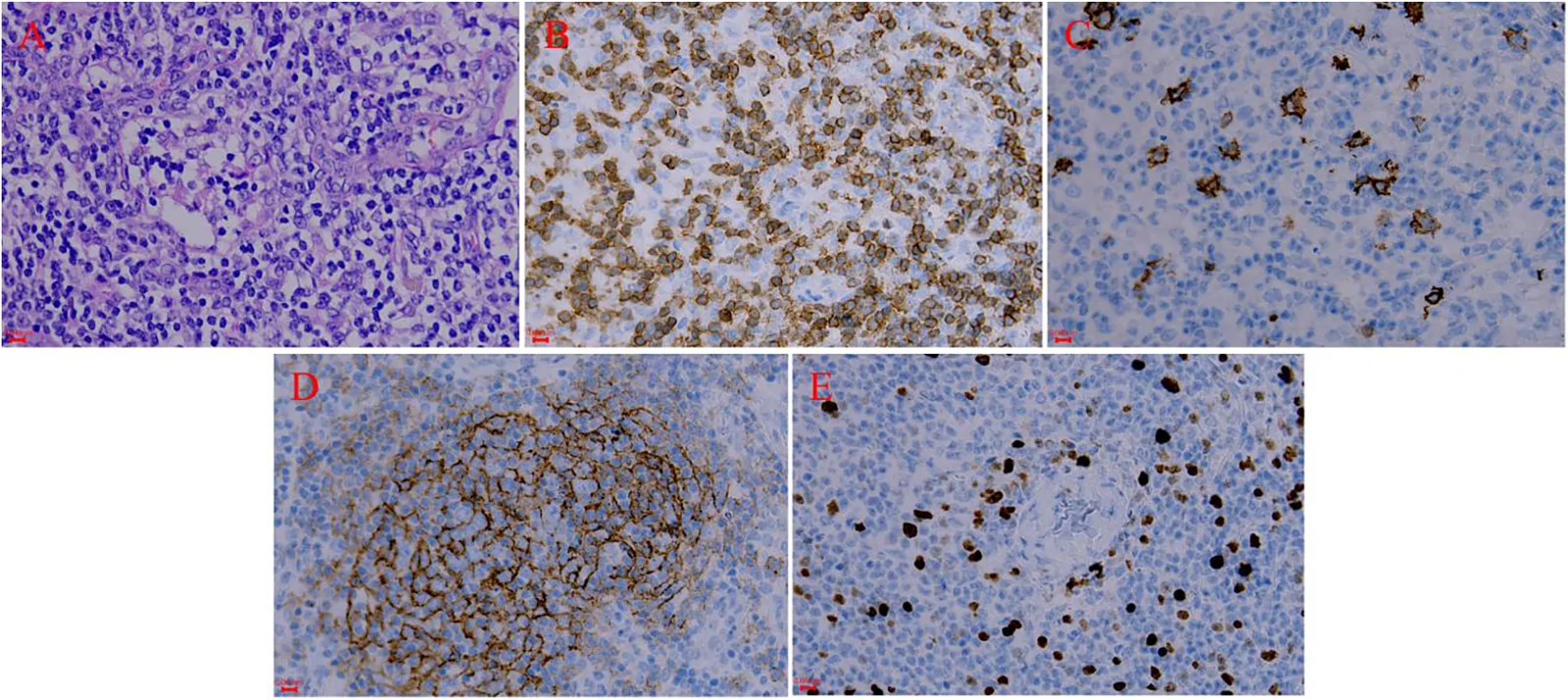
Histopathological findings from the right inguinal lymph node biopsy. **(A)** Hematoxylin and eosin staining shows reactive lymphoid hyperplasia. **(B)** CD3-positive T-cell areas. **(C)** CD20-positive B-cell areas. **(D)** CD21-positive follicular dendritic cell meshwork. **(E)** Ki-67 staining.

**Table 2:** Yamaguchi classification criteria for AOSD and the features fulfilled by this patient.

Yamaguchi criterion	Major/minor	Fulfilled in this patient
Fever of ≥39 °C for ≥1 week	Major	Yes (peak 39.6 °C; fever >3 weeks)
Arthralgia or arthritis for ≥2 weeks	Major	No (no arthritis observed)
Typical evanescent rash	Major	Yes (diffuse erythematous rash)
Leukocytosis ≥10,000/µL with ≥80% neutrophils	Major	No (highest WBC 9.45 × 10⁹/L during hospitalization; mild leukocytosis reported at the referral hospital)
Sore throat	Minor	Yes
Lymphadenopathy and/or splenomegaly	Minor	Yes (generalized lymphadenopathy and splenomegaly)
Abnormal liver function tests	Minor	Yes (elevated transaminases and GGT)
Negative rheumatoid factor and ANA	Minor	Yes (RF and ANA negative)
Diagnosis: ≥5 criteria including ≥2 major criteria	-	Fulfilled (2 major + 4 minor criteria)

### 2.4 Therapeutic intervention

The initial dose of intravenous methylprednisolone was 80 mg per day (approximately 1.2 mg/kg/day; body weight was 65 kg), which was reduced to 40 mg/day, then to prednisone 40 mg/day, and finally to methylprednisolone 32 mg/day before discharge. Supportive treatments included gastric mucosal protection, calcium supplementation, and liver protection therapy. No biological agents were used during this hospital stay. After thoroughly investigating the potential for major infections and malignancies, and particularly after lymph node pathology did not support lymphoma, high-dose glucocorticoid therapy was initiated.

### 2.5 Follow-up and outcomes

The clinical response was swift. The fever subsided, the diffuse rash gradually faded, and the itching disappeared (Figure 1D). A series of quantitative data reconstructed from the original medical records was plotted according to available sampling dates or time points. The temperature curve indicated a drop from a fever exceeding 39 °C to a consistently normal body temperature during hospitalization. Inflammatory markers showed a similar trend: ferritin levels decreased from 15,485 ng/mL to 8,496, 2,070, 1,438, and 712 ng/mL during hospitalization, ultimately reaching 318 ng/mL at follow-up; high-sensitivity C-reactive protein fell from a peak of 74.97 mg/L to 0.28 mg/L before discharge; and procalcitonin decreased from 0.514 to <0.020 ng/mL. Hemoglobin levels recovered from 102 to 121 g/L at discharge and 136 g/L at the most recent follow-up. These changes are illustrated in Figure 3. Liver enzymes, which had been markedly elevated during hospitalization [alanine aminotransferase (ALT) 169 U/L and gamma-glutamyl transferase 273 U/L], decreased to 28 U/L and 56 U/L, respectively, during treatment and follow-up. Overall clinical and biochemical trends showed a steroid-responsive inflammatory disease rather than uncontrolled infection or lymphoma. After stabilization, the patient was discharged with planned regular outpatient follow-up. At discharge, the fever had resolved and the rash had improved significantly. During 5 months of follow-up after discharge, the patient remained free of fever and rash recurrence, corticosteroids were tapered successfully, and no disease-modifying antirheumatic drugs (DMARDs) or biologic agents were required.

**Figure 3:**
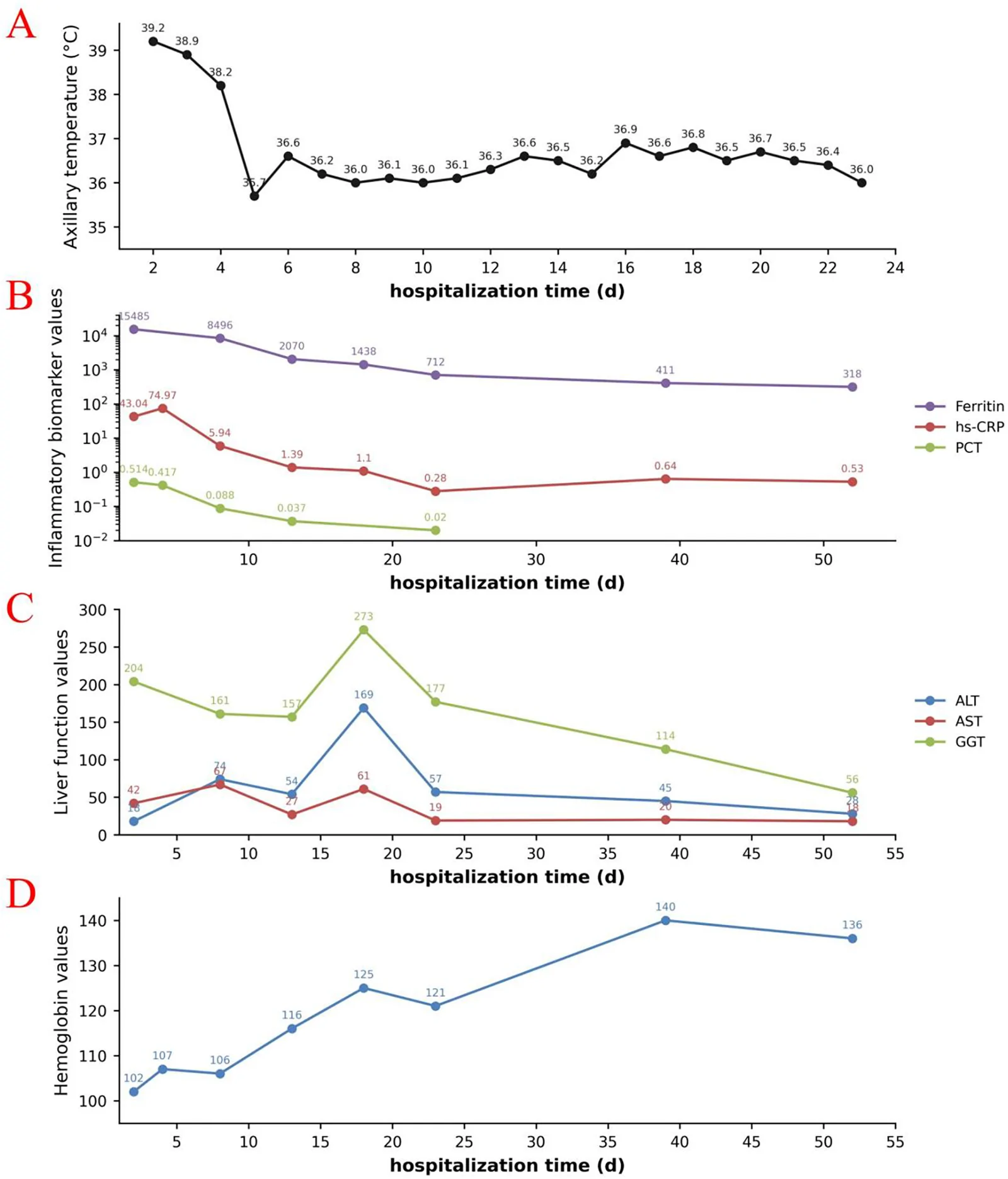
Clinical course and longitudinal laboratory trends. **(A)** Axillary temperature over time. **(B)** Normalized trajectories of inflammatory biomarkers, including ferritin, high-sensitivity C-reactive protein, and procalcitonin. **(C)** Serial liver enzyme measurements, including ALT, AST, and GGT. **(D)** Hemoglobin trajectory during treatment and follow-up.

## 3 Discussion

A case such as this, with extensive lymphadenopathy, splenomegaly, and high metabolic activity on 18F-FDG PET/CT, is uncommon in AOSD (6, 14). These “lymphoma-like” features, such as diffuse bone marrow metabolic activity, often complicate the differential diagnosis between AOSD and lymphoma (5). There are many reports of AOSD cases with similar imaging findings in which lymphoma was strongly suspected but was subsequently excluded by lymph node biopsy (4, 14, 15). What distinguishes this case from the majority of reported cases is the extremely elevated serum ferritin level of 15,485 ng/mL. Hyperferritinemia is a characteristic of AOSD (11, 16, 17), but reaching such extreme levels (>15,000 ng/mL) is relatively rare in the literature and therefore represents an important diagnostic clue. Studies have suggested that an extremely high ferritin level may be associated with more extensive organ involvement and greater disease activity (18).

Comparisons with previously published cases highlight the distinctive features of this patient. Lymphoma-like AOSD with generalized lymphadenopathy and PET-positive disease has been described (4, 6, 14, 15), and a systematic review identified cases with concurrent AOSD and lymphoma (19). However, only a limited number of reports describe AOSD with serum ferritin levels exceeding 15,000 ng/mL in the setting of PET-positive generalized lymphadenopathy; the combination of extreme hyperferritinemia, generalized lymphadenopathy, and diffuse bone marrow metabolic activity makes this case instructive for clinicians (11, 14, 15).

We decided not to rely on clinical features but on the principle of “exclude first, diagnose later.” Unlike other reported cases that rely on the metabolic features of PET/CT and clinical features, we performed an excisional biopsy even when PET/CT strongly suggested lymphoma. Histopathology, immunohistochemistry, and T-cell receptor gene rearrangement tests in the right inguinal lymph node showed no evidence of clonality, providing a clear pathological basis to rule out lymphoma because the metabolic features of PET/CT alone cannot reliably distinguish the inflammatory lymphadenopathy of AOSD from lymphoma (20). This patient also had a rapid response to the high-dose glucocorticoid therapy, with rapid normalization of body temperature, resolution of the rash, regression of lymphadenopathy, and marked decreases in serum ferritin and C-reactive protein. This series of responses also supports the diagnosis of AOSD and aligns with other reports of AOSD cases responding well to corticosteroid treatment (21, 22). Beyond lymphoma, other conditions presenting with fever, generalized lymphadenopathy, and extreme hyperferritinemia should be considered, including hemophagocytic lymphohistiocytosis/macrophage activation syndrome and rare entities such as intravascular large B-cell lymphoma, which can be revealed by a hemophagocytic syndrome (23), and TAFRO syndrome, characterized by thrombocytopenia, anasarca, fever, reticulin fibrosis, and organomegaly (24). These diagnoses were actively considered in our patient; however, a bone marrow examination was not performed during the hospitalization.

This case illustrates several important educational points. First, differential diagnosis of AOSD can be difficult when primary symptoms include significant lymphadenopathy, splenomegaly, and diffuse hypermetabolism on PET/CT scans (4, 15). Second, even when imaging strongly suggests malignancy, lymph node biopsy is still the “gold standard” for differential diagnosis and cannot be replaced by non-invasive tests. Pathological features of lymph nodes in AOSD typically exhibit reactive hyperplasia (mixed lymphoid or sinus histiocytosis), whereas lymphoma is characterized by monoclonal proliferation and architectural disruption, a distinction that is crucial for differentiation (19). Furthermore, infectious causes of generalized lymphadenopathy and fever, including tuberculosis (particularly in endemic regions), cat scratch disease (*Bartonella henselae*), and infectious mononucleosis (Epstein–Barr virus), should be considered in the differential diagnosis (7, 8, 10). Third, the mechanisms underlying extremely high ferritin levels (>10,000 ng/mL) deserve further investigation. While AOSD can cause hyperferritinemia, high ferritin levels may indicate subclinical MAS or a “cytokine storm” (11, 17). Although ferritin is a useful diagnostic marker of AOSD, marked hyperferritinemia is not specific and may also be observed in infections, malignancies, and hemophagocytic syndromes (10, 11); in one comparative study, ferritin levels were significantly higher in AOSD than in sepsis (25). From a treatment point of view, the rapid response to high-dose glucocorticoids confirmed the diagnosis and indicated that even severe systemic inflammation resembling lymphoma may be steroid-sensitive. This is in line with other reports of AOSD showing an initial response to first-line corticosteroids, although relapse or the need for additional immunomodulatory agents is common (26, 27). Clinicians should be vigilant when encountering patients with unexplained fever, widespread lymphadenopathy, and extremely high serum ferritin levels. Excisional biopsy combined with serum ferritin analysis can help avoid misdiagnosis and inappropriate treatment.

This case provides practical guidance for the management of AOSD presenting with “lymphoma-like” features. When a patient presents with unexplained fever, generalized lymphadenopathy, splenomegaly, markedly elevated ferritin levels, and 18F-FDG PET/CT findings suggestive of lymphoma, lymph node biopsy is needed to avoid misdiagnosis and inappropriate treatment (e.g., chemotherapy). In this case, the diagnosis was established on the basis of lymph node pathology, immunohistochemistry, gene rearrangement analysis, the extremely elevated ferritin level, and the rapid response to corticosteroids. This case reinforces the diagnostic principle of “exclude first, diagnose later” and hyperferritinemia proved to be an important clue for clinical decision-making.

Because of the markedly elevated ferritin level (15,485 ng/mL), MAS/HLH was considered in the differential diagnosis. The HLH-2004 diagnostic criteria include fever, splenomegaly, cytopenias, hypertriglyceridemia or hypofibrinogenemia, hemophagocytosis in the bone marrow, low or absent natural killer (NK)-cell activity, elevated soluble CD25, and elevated ferritin (28). In this patient, fibrinogen was elevated (5.26 g/L), D-dimer was markedly elevated at admission (5.23 mg/L, decreasing to 0.79 mg/L), and triglycerides were 1.92 mmol/L (reference <1.7 mmol/L); however, NK-cell activity, soluble CD25, and bone marrow examination were not performed during the hospitalization, which should be acknowledged as a limitation. The absence of significant cytopenias, coagulopathy, and progressive organ failure, together with the rapid and sustained response to glucocorticoids, made fulminant MAS/HLH unlikely, although subclinical MAS could not be entirely excluded.

The antecedent SARS-CoV-2 infection in this patient also merits discussion. Viral infections, including SARS-CoV-2, have been reported as potential triggers of AOSD (29). Although a causal relationship cannot be established in a single case, clinicians should be aware that AOSD may emerge in the context of a preceding COVID-19 illness and that persistent fever, rash, and extreme hyperferritinemia should not be dismissed as residual viral symptoms.

The transient evanescent rash was an important diagnostic clue in this patient. Cutaneous manifestations are recognized as having significant diagnostic value in AOSD, and recognition of the typical evanescent rash may facilitate earlier diagnosis and avoid unnecessary invasive investigation (30). This case report was prepared in accordance with the CARE (CAse REport) guidelines (31).

However, this report has limitations. As a single case, it cannot fully reflect the heterogeneity of AOSD. Although the patient remained relapse-free for 5 months after discharge, with successful corticosteroid tapering and no requirement for DMARDs or biologic agents, longer follow-up is still needed to fully evaluate the risk of relapse and the long-term need for immunosuppressive therapy. Further cohort studies or case series are needed to clarify the diagnostic and prognostic value of extreme hyperferritinemia in AOSD. Overall, combining lymph node biopsy with ferritin assessment may help distinguish AOSD from lymphoma-like diseases and support early, accurate treatment.

## Patient perspective

A separate patient perspective statement was not available; written informed consent for publication was obtained.

## Data Availability

The raw data supporting the conclusions of this article will be made available by the authors, without undue reservation.
